# Supplementary material for: Tumor-Suppressor Role of the α1-Na/K-ATPase Signalosome in NASH Related Hepatocellular Carcinoma
Source: Int J Mol Sci. 2022 Jul 1;23(13):7359. doi: 10.3390/ijms23137359 (PMC9266688; doi:10.3390/ijms23137359)
Supplement: Supplementary file 1 [file ijms-23-07359-s001.zip › ijms-1783267-supplementary.pdf]

Supplementary Material

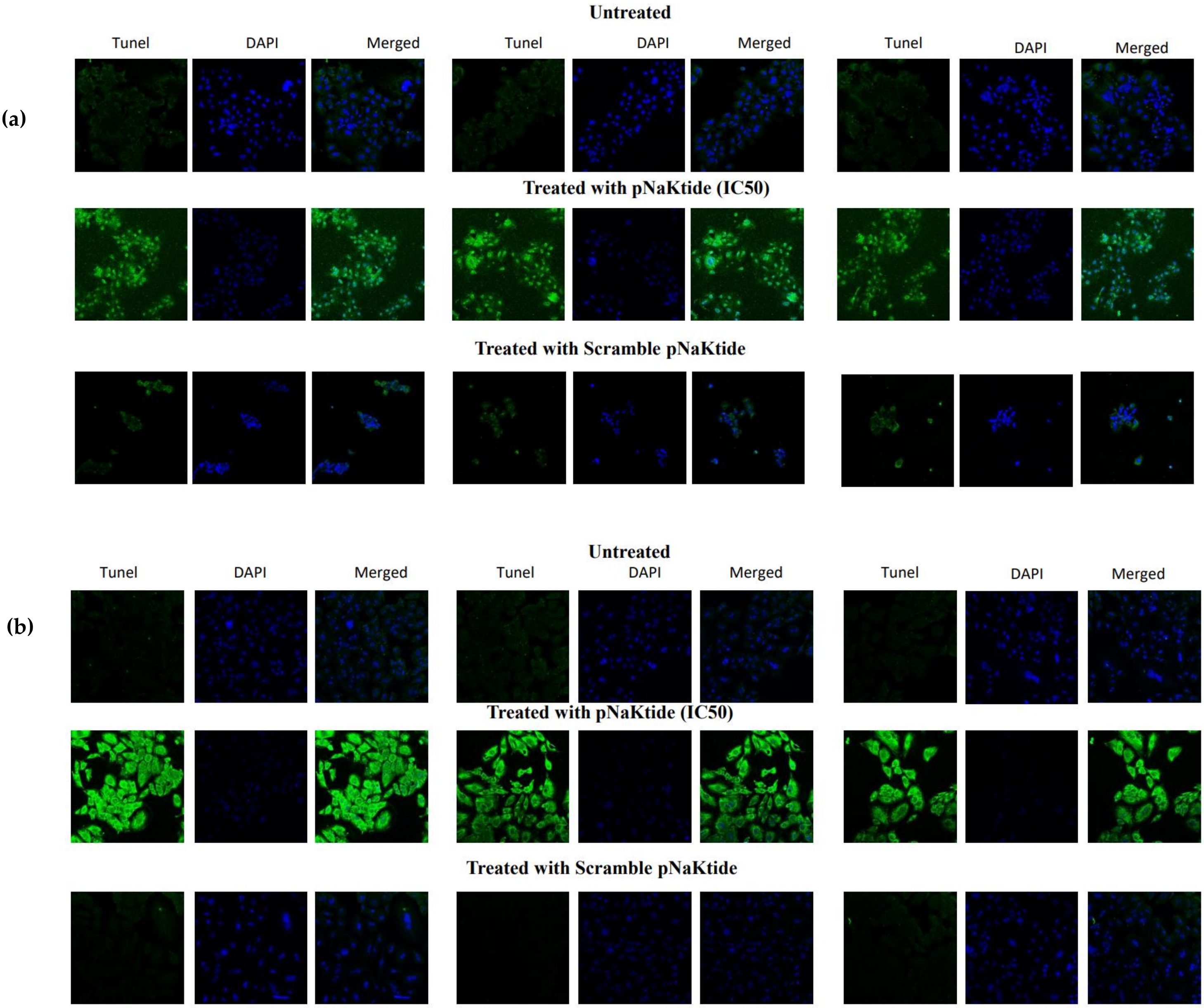

Figure S1. (**a**) Confocal images of pNaKtide induced apoptosis in Hep3B cell lines with Scramble pNaKtide as controls. (**b**) Confocal images of pNaKtide induced apoptosis in SNU475 cell lines with Scramble pNaKtide as controls. Magnification ×20.

(a)

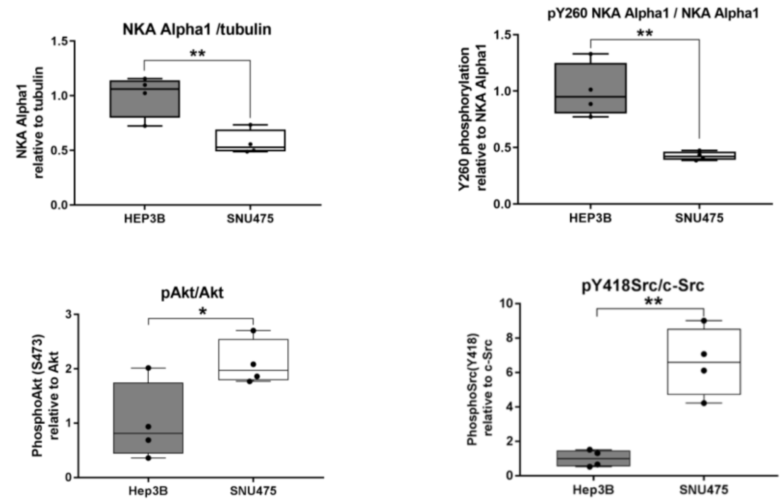

(b)

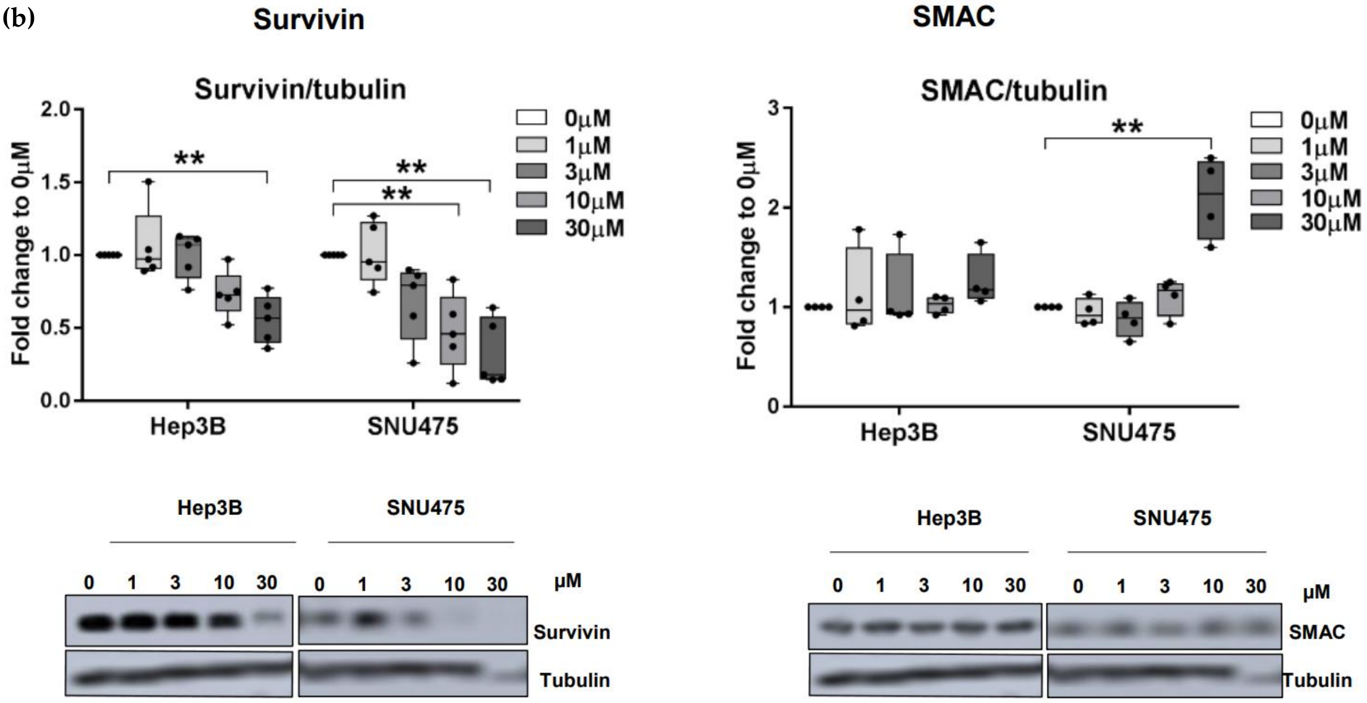

Figure S2. (**a**) Basal expression of *α1-NKA, pSrc, pAkt* and *pY260* in Hep3Band SNU475 cell lines. Baseline α1-NKA and pY260 expression was lower in SNU475 cell line than Hep3B cell line. On the contrary, there was a high baseline Src and Akt phosphorylation in SNU475 cell line than Hep3B cell line ($n = 4$, $^{**}p < 0.01$ by ANOVA and Turkey's Post hoc test). (**b**) *Survivin* and *SMAC* dose-response to pNaKtide treatment in two HCC cell lines. There was a progressive downregulation of survivin with concomitant SMAC protein upregulation in both human HCC cell lines following increased pNaKtide administration, with upregulation of SMAC expression especially at the highest pNaKtide dose**.** Dose-response on targeted protein expression, survivin & SMAC in Hep3B and SNU475 cell lines exposed to pNaKtide (0,1, 3, 10 and 30 μM) up to 18 hours. Results are shown as box-whisker plots showing a fold change in each protein relative to 0 μM. (n = 4–5 for each group, ** $p < 0.01$ by ANOVA and Turkey's Post hoc test).

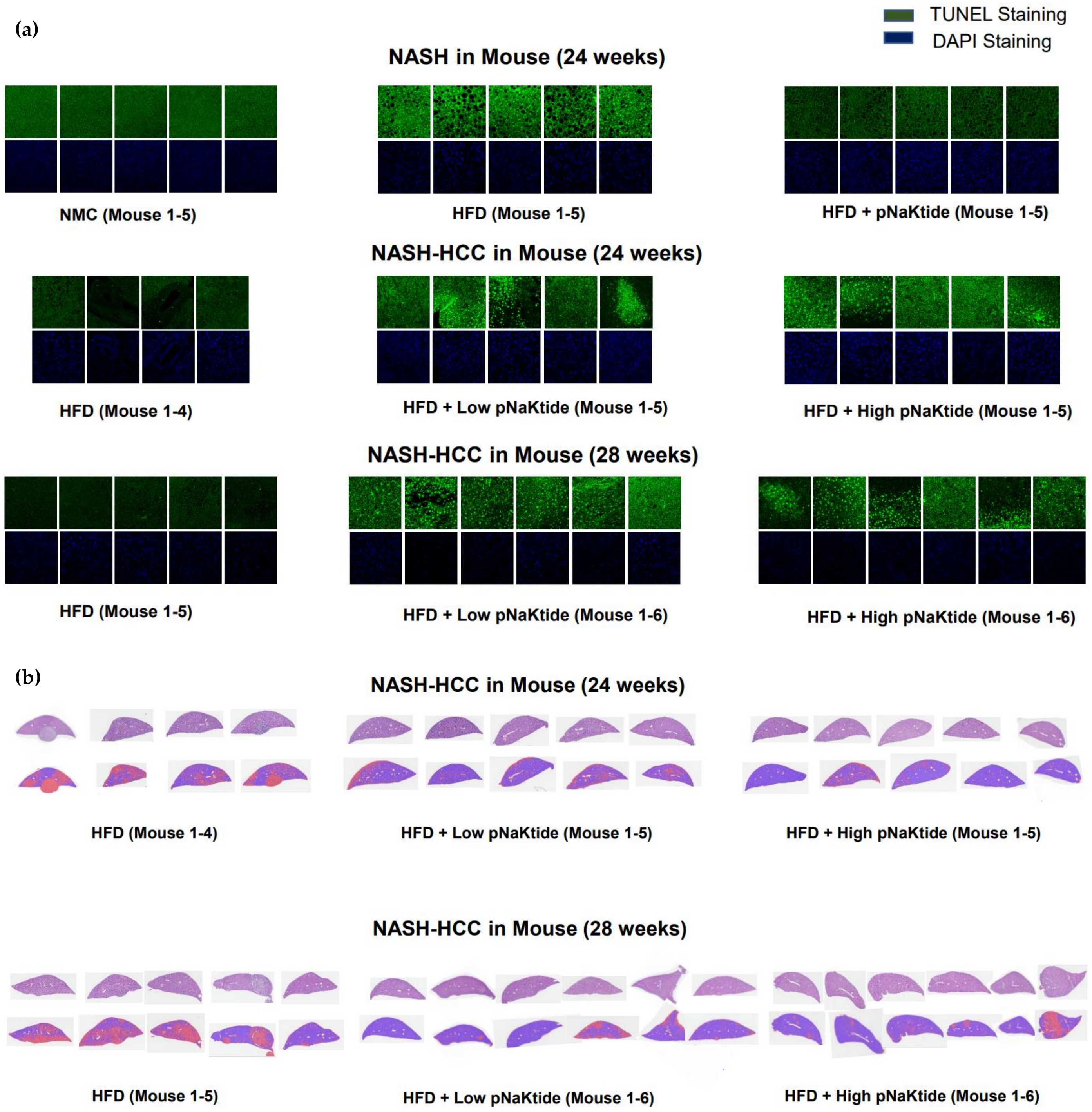

Figure S3. In vivo evaluation of α1-NKA Signalosome normalization on NASH and NASH related HCC at 24 (early) and 28 weeks (advanced) models. (**a**) Representative confocal images showing apoptotic cells (TUNEL assay, positive for apoptosis cell in green) in liver tissue/tumor cells of each mouse per experimental group of both NASH (24 weeks) and NASH-HCC (24 and 28 weeks) murine models. (**b**) Tumor Burden was evaluated on H&E-stained slides at low magnification of each mouse per experimental group of both the 24 and 28 weeks NASH-HCC murine models.

(a)

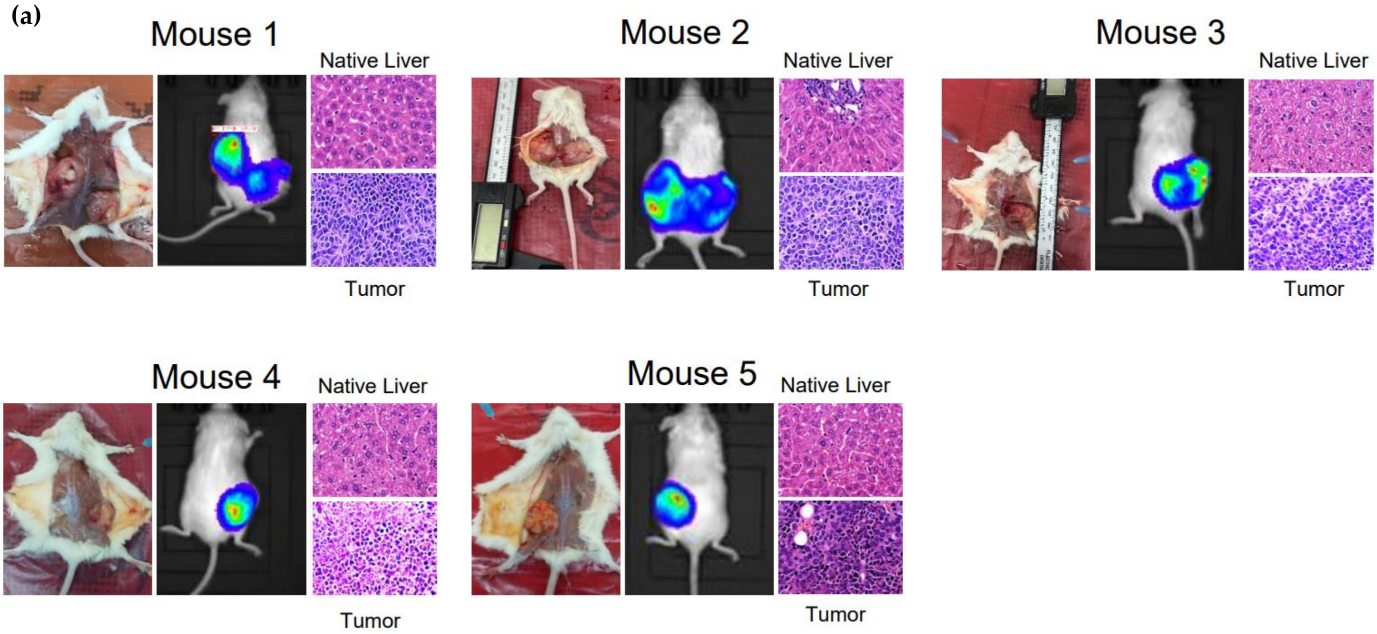

Mouse 1
Native Liver
Tumor
Mouse 2
Native Liver
Tumor
Mouse 3
Native Liver
Tumor
Mouse 4
Native Liver
Tumor
Mouse 5
Native Liver
Tumor

(b)

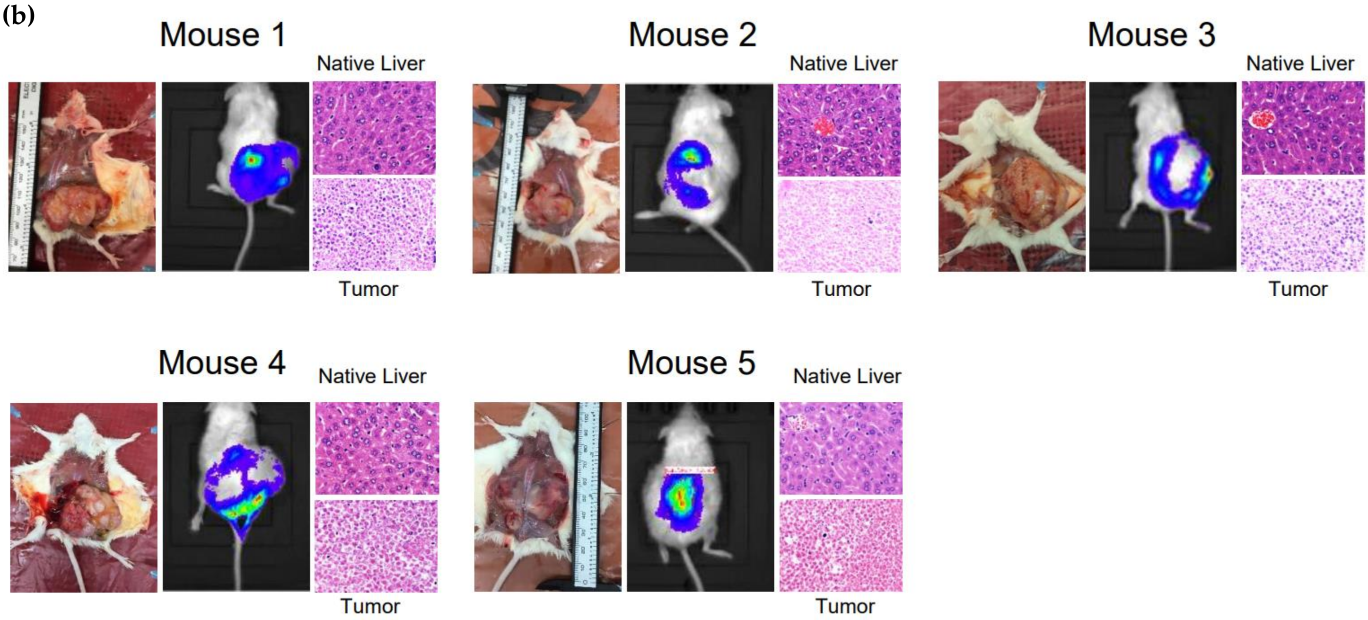

Mouse 1
Native Liver
Tumor
Mouse 2
Native Liver
Tumor
Mouse 3
Native Liver
Tumor
Mouse 4
Native Liver
Tumor
Mouse 5
Native Liver
Tumor

(c)

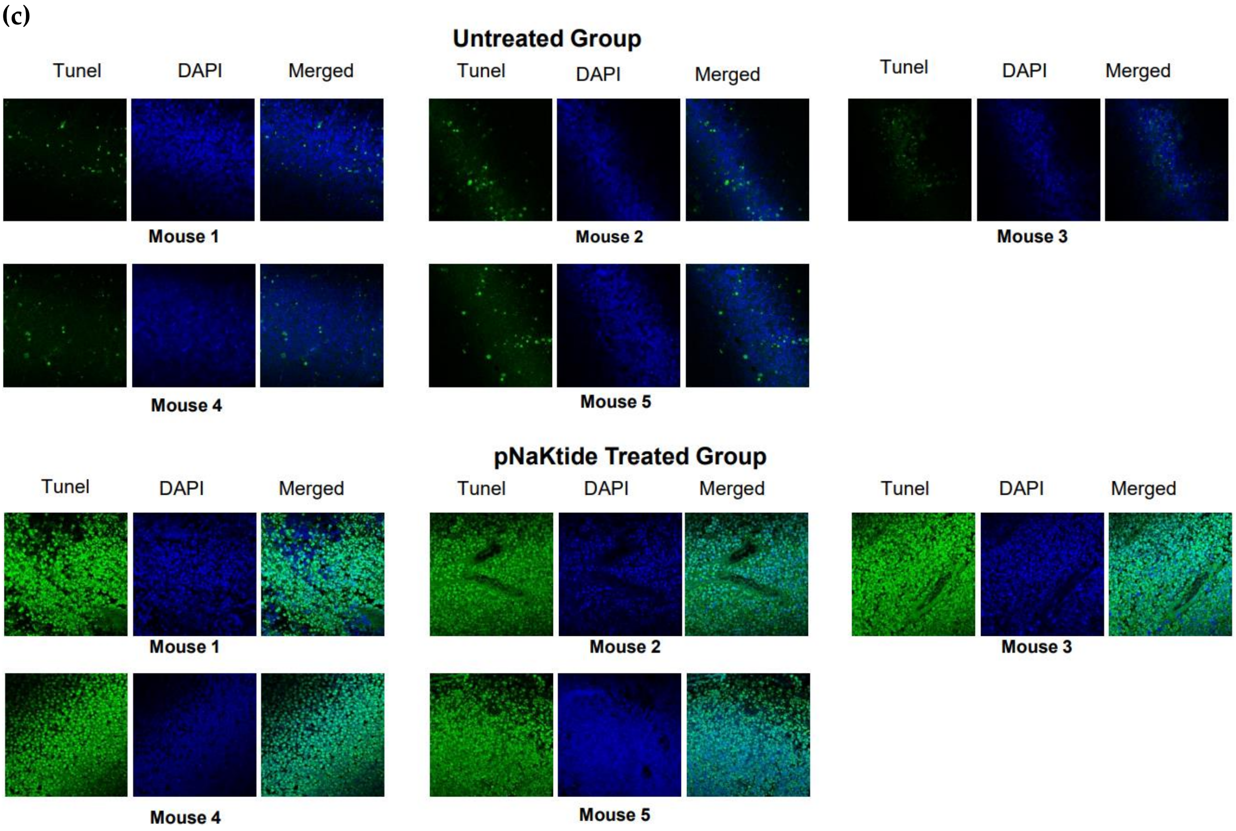

(d)

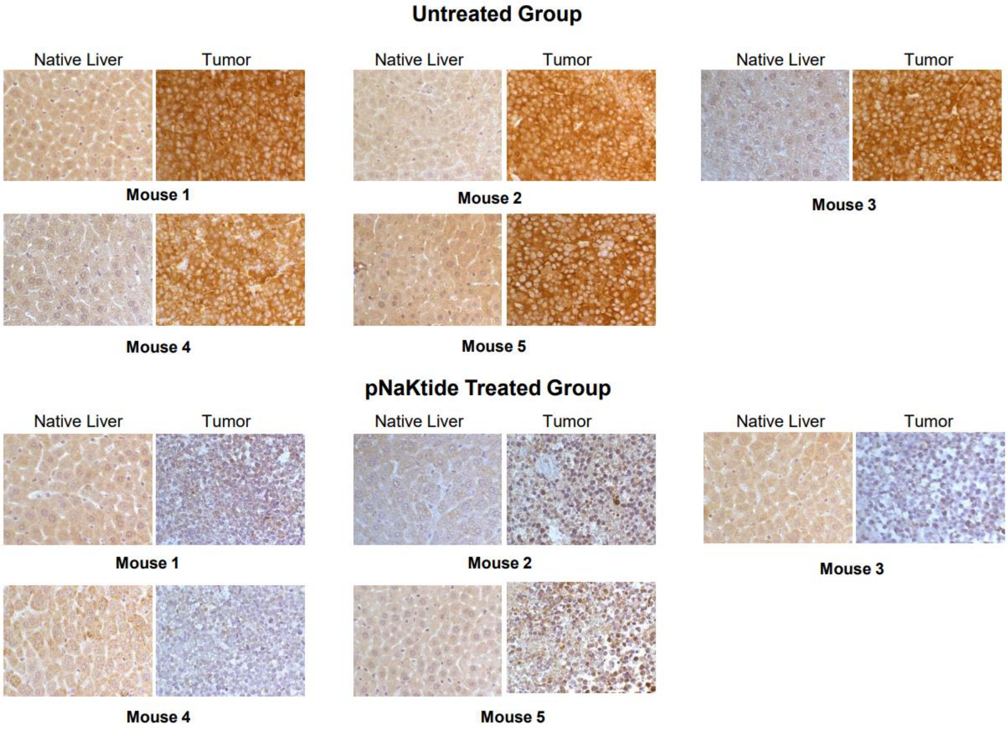

Figure S4. HCC analysis, apoptosis and Survivin expression in SCID MICE xenograft tumors. (**a**) Representative images of xenograft tumors, IVIS—videoed luminescence of tumors and H&E-stained microscopy images of liver and tumor sections for each of the mouse in the untreated group. (**b**) Representative images of xenograft tumors, IVIS—videoed luminescence of tumors and H&E-stained microscopy images of liver and tumor sections for each of the mouse in the pNaKtide group. (**c**) Representative confocal images showing apoptotic cells (TUNEL assay, positive for apoptosis cell in green, nuclei in blue; DAPI) in xenograft tumor cells of each mouse in the untreated and pNaKtide treated groups. (**d**) Representative images of survivin expression in liver tissues and xenograft tumor cells from each mouse in the untreated and pNaKtide treated groups.

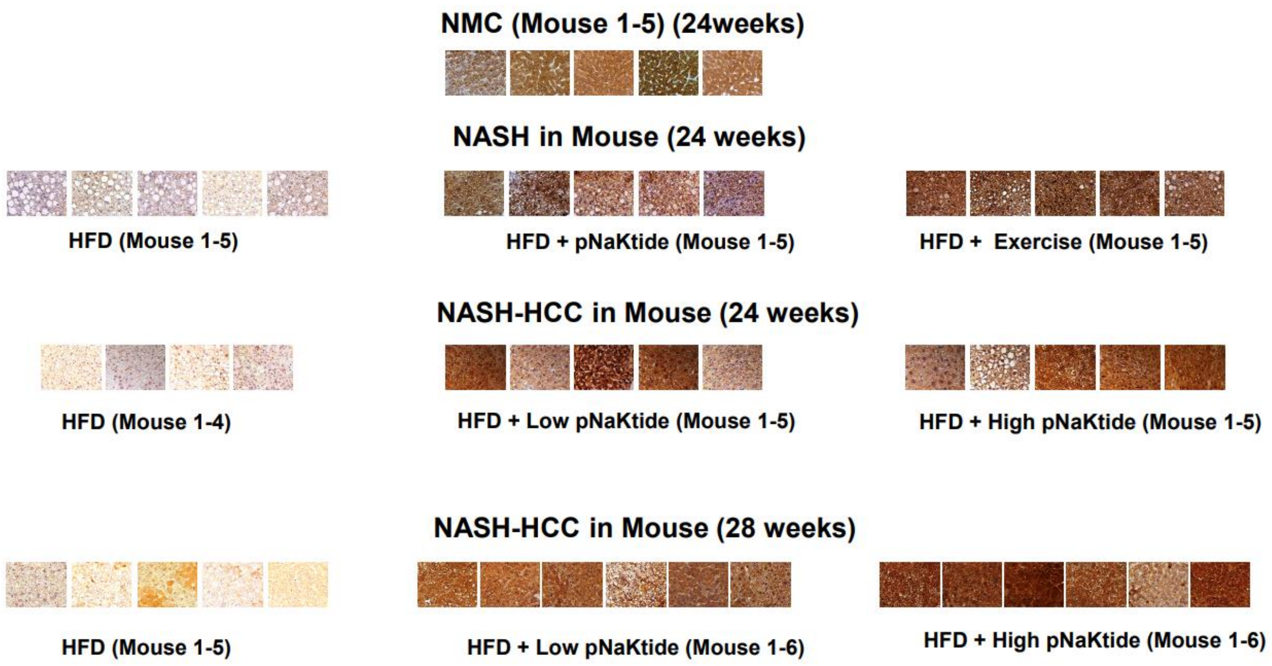
NMC (Mouse 1-5) (24weeks)
NASH in Mouse (24 weeks)
HFD (Mouse 1-5)
HFD + pNaKtide (Mouse 1-5)
HFD + Exercise (Mouse 1-5)
NASH-HCC in Mouse (24 weeks)
HFD (Mouse 1-4)
HFD + Low pNaKtide (Mouse 1-5)
HFD + High pNaKtide (Mouse 1-5)
NASH-HCC in Mouse (28 weeks)
HFD (Mouse 1-5)
HFD + Low pNaKtide (Mouse 1-6)
HFD + High pNaKtide (Mouse 1-6)

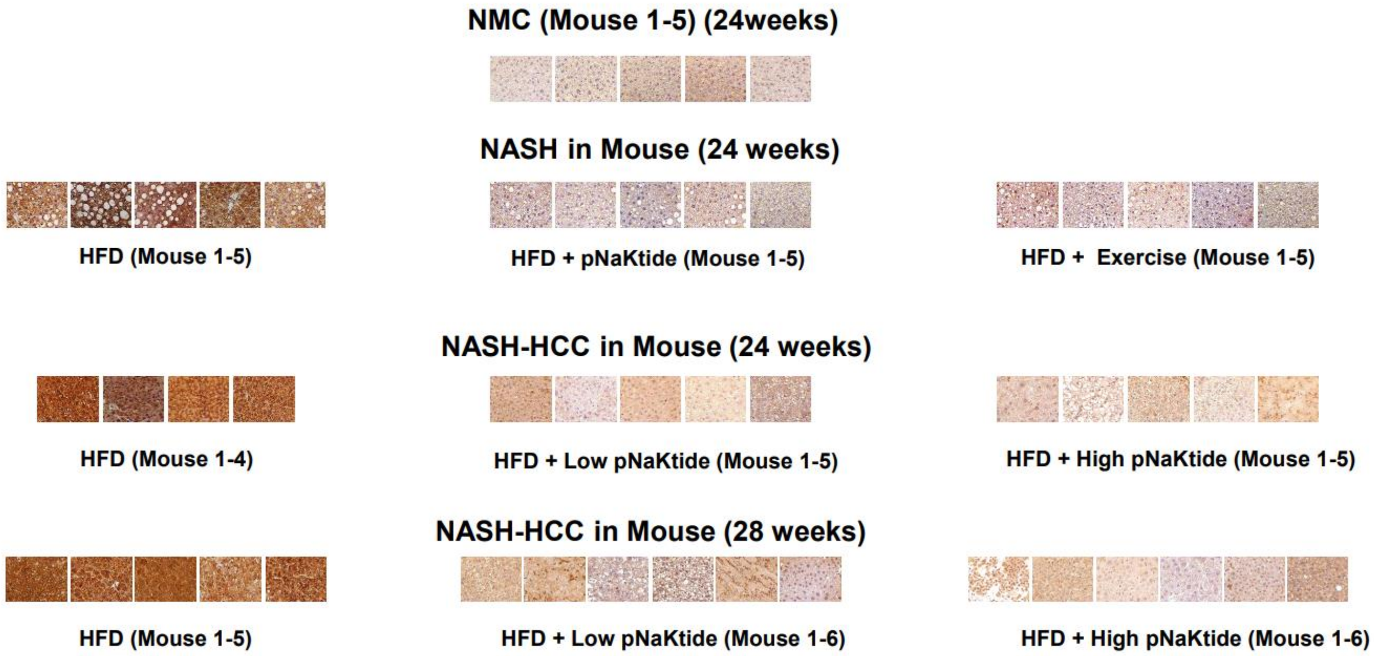
NMC (Mouse 1-5) (24weeks)
NASH in Mouse (24 weeks)
HFD (Mouse 1-5)
HFD + pNaKtide (Mouse 1-5)
HFD + Exercise (Mouse 1-5)
NASH-HCC in Mouse (24 weeks)
HFD (Mouse 1-4)
HFD + Low pNaKtide (Mouse 1-5)
HFD + High pNaKtide (Mouse 1-5)
NASH-HCC in Mouse (28 weeks)
HFD (Mouse 1-5)
HFD + Low pNaKtide (Mouse 1-6)
HFD + High pNaKtide (Mouse 1-6)

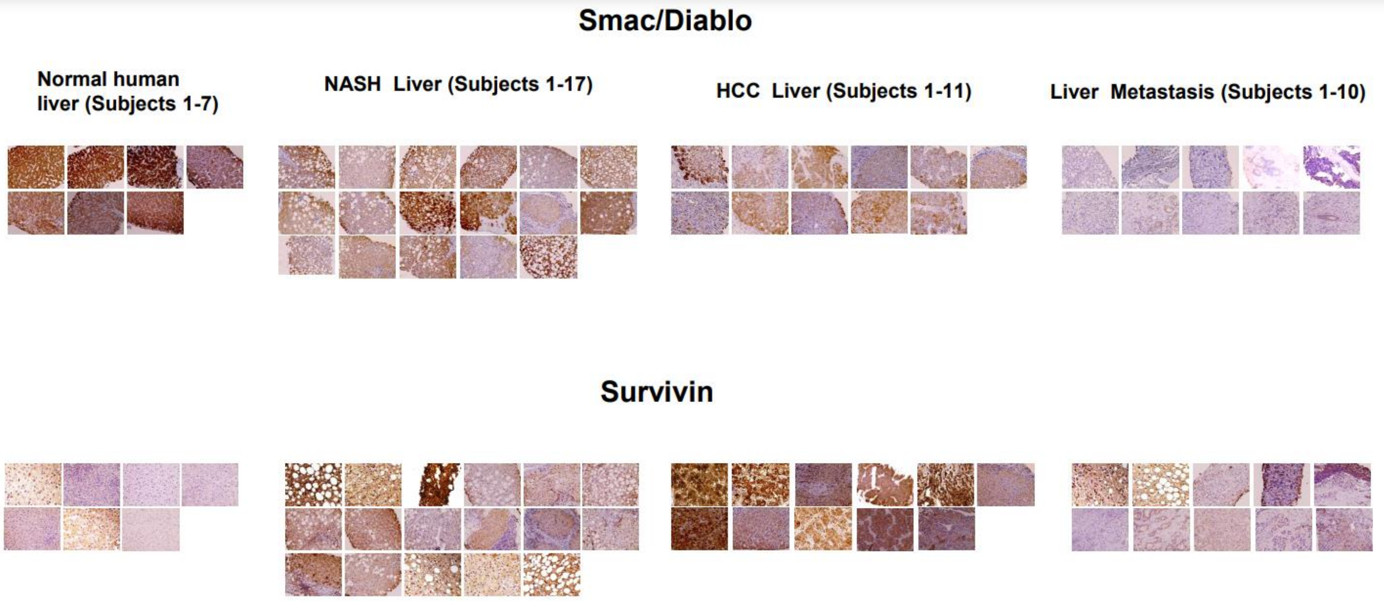

Figure S5. Protein expression by confocal immunostaining of both NASH (24 weeks) and NASH-HCC (24 and 28 weeks) murine models and human subjects. (**a**). Representative images of Smac immunostaining on liver tissues/tumor cells from each animal used for quantitation of Smac per experimental group (**b**) Representative images of survivin immunostaining on liver tissues/tumor cells from each mouse used for quantitation of survivin per experimental group. (**c**) Representative images of SMAC/Diablo and survivin immunostaining on human liver tissues from individual patients with the diagnosis of NASH, HCC, liver secondary malignancies (metastases) and normal subjects included for quantitation.

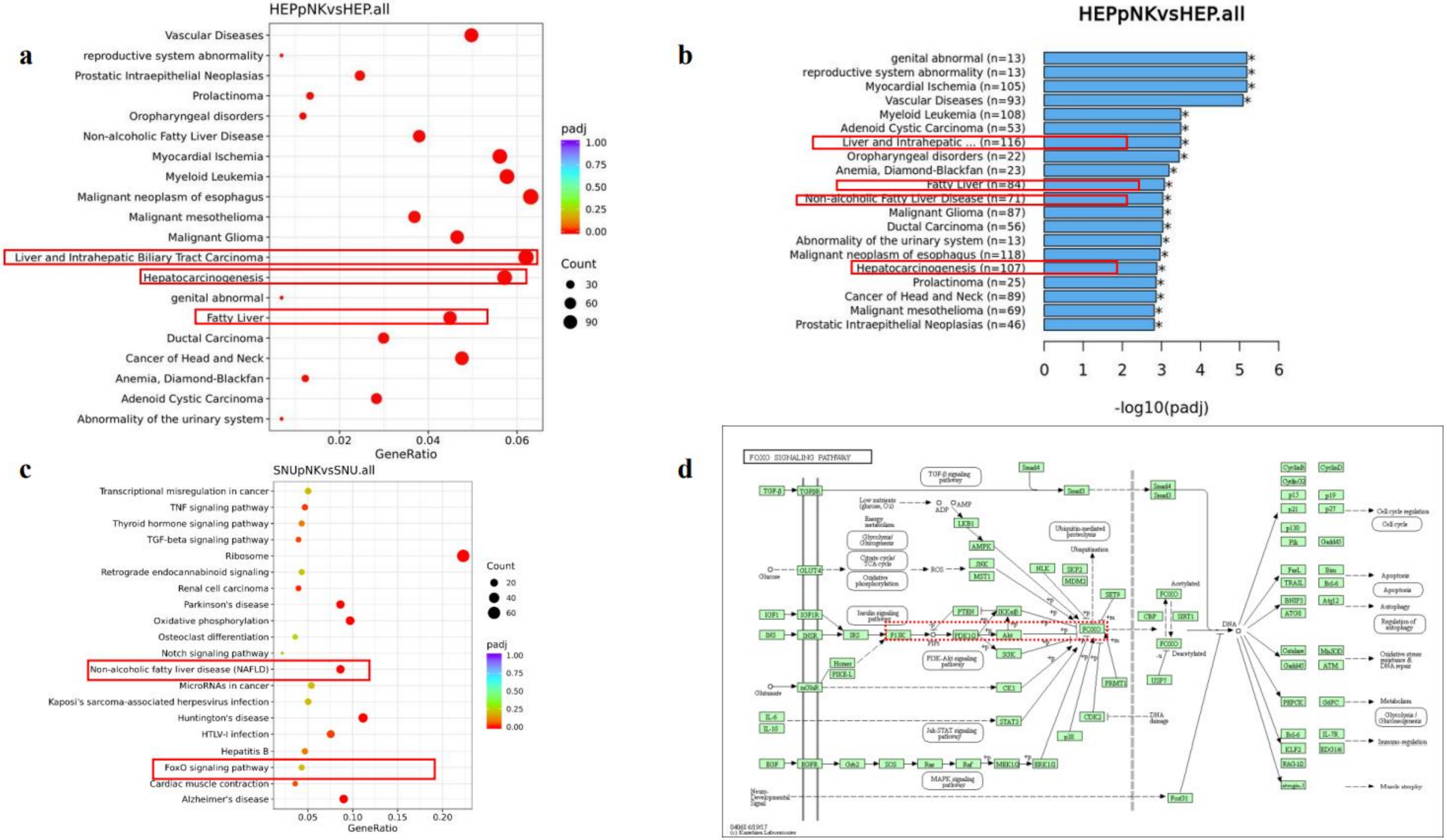

Figure S6. Extended RNA sequencing analysis data from Human HCC cell lines. (**a**,**b**) Dots plots and bars showing liver and related diseases pathways that are activated in HCC cell lines and ameliorated via α1-NKA Signalosome normalization by pNaKtide. (**c**) Dots plots showing the enrichment of FOXO signaling pathway. (**d**) RNA sequencing KEGG diagram showing the enrichment of the FOXO signaling pathway.

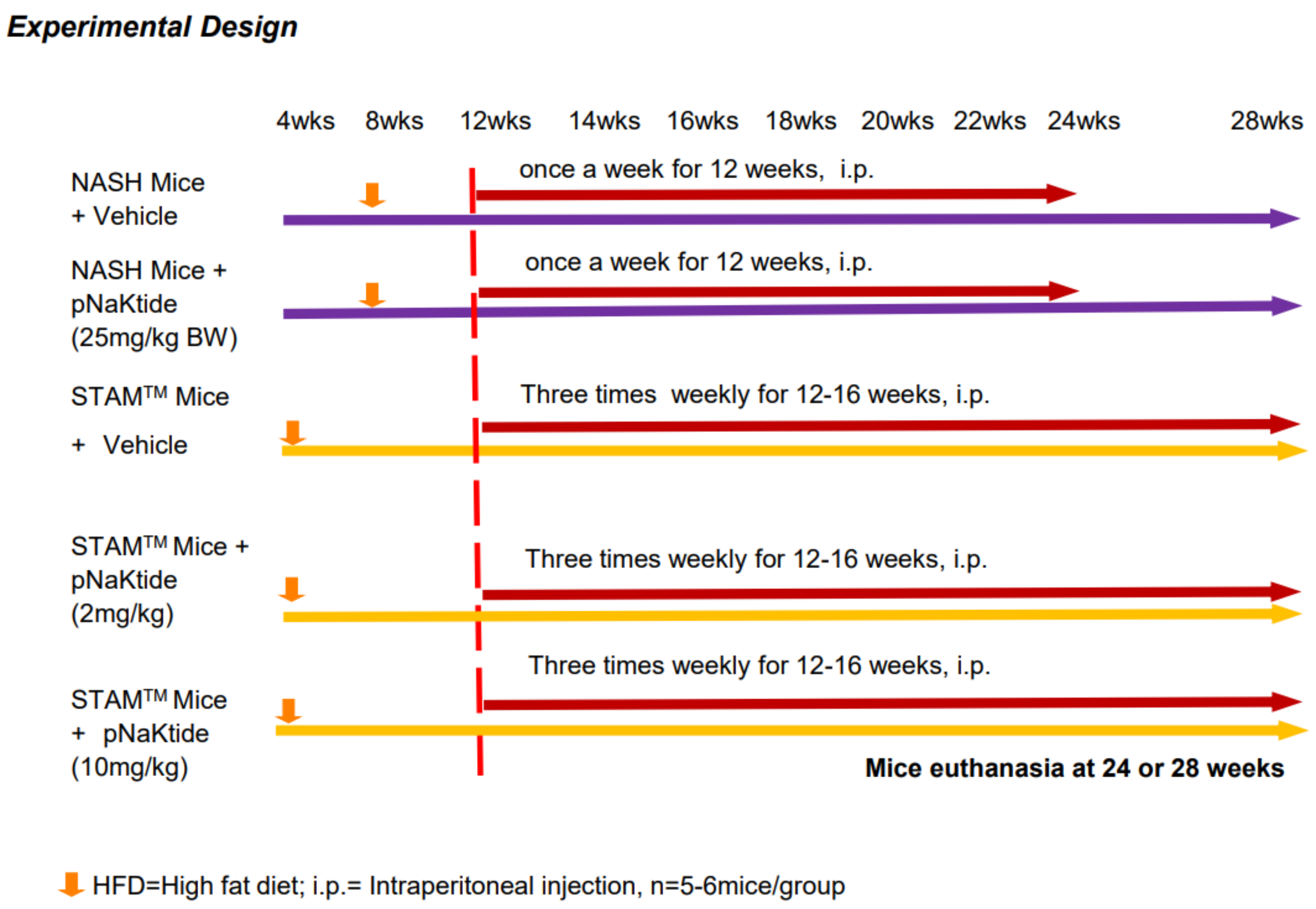

Figure S7. Chart showing the experimental design for NASH and NASH-HCC mouse model. The chart displays the timeline and interventions for the NASH and NASH-HCC mouse model experiments. The pNaKtide treated group in the NASH experiment received a single dose of pNaKtide (25mg/kg BW, i.p.) while the mice in the NASH-HCC model received varying doses pNaKtide (2mg/kg BW, low dose, i.p) or (10mg/kg BW, high dose, i.p), thrice every week. All mice were placed on HFD ad libitum throughout the experimental period that lasted 24 weeks for the NASH experiment and early-stage HCC arm (12 weeks of treatment), or 28 weeks for the late-stage NASH-HCC experiment (16 weeks of treatment).

Table S1. Key reagents and chemical used in this study. Antibodies against the indicated protein as well as other reagents/chemicals used in this study are as listed below. Their catalogue number, source and the dilutions used in the experiments are as listed.

ANTIBODY/REAGENT/CHEMICAL	Source	Reference	Description	Host Sp.	IHC	ICC	WB
Phospho-Src	Thermofisher Scientific	Catt 44- 660G	Polyclonal	Rabbit	N/A	1:100	1:1000
c-Src	Santa Cruz Biotechnology	Catt Sc-8056	Monoclonal	Mouse	N/A	N/A	1:1000
Smac/Diablo	Abcam	Catt ab8115	Polyclonal	Rabbit	1:200	1:100	1:1000
Rabbit specific HRP/DAB Detection IHC kit- Micro-polymer	Abcam	Catt ab236469	Micro-Polymer	Goat	N/A	N/A	N/A
Survivin	abcam	Catt ab469	Polyclonal	Rabbit	1:500	1:250	1:1000
Goat Anti-Rabbit IgG H&L (Alexa Fluor® 594)	Abcam	Catt ab150080	Polyclonal Secondary	Goat	1:500	1:750	N/A
Goat Anti-Rabbit IgG H&L (Alexa Fluor® 488), preabsorbed	Abcam	Catt ab150081	Polyclonal Secondary	Goat	N/A	1:750	N/A
alpha 1 Na/K-ATPase	Millipore	Catt 05-369	Monoclonal primary	Mouse	N/A	1:100	N/A
alpha tubulin	Sigma-Aldrich	Catt T5168	Monoclonal primary	Mouse	N/A	1:100	1:5000
Mouse monoclonal antibody anti Na/K-ATPase (α6f)	Developmental Studies- University of Iowa	N/A	Monoclonal primary	Mouse	N/A	N/A	1:1000
Rabbit monoclonal anti phospho ERK1/2(MAPK) antibody	Cell Signaling Technologies	Catt 9154	Monoclonal primary	Rabbit	N/A	N/A	1:1000
Mouse Monoclonal β- Catenin antibody	BD Biosciences	Catt BDB610154	Monoclonal primary	Mouse	N/A	N/A	1:1000

ANTIBODY/REAGENT/CHEMICAL	Source	Reference	Description	Host Sp.	IHC	ICC	WB
Rabbit monoclonal anti ERK1/2(MAPK) antibody	Cell Signaling Technologies	Catt . 4694	Monoclonal primary	Rabbit	N/A	N/A	1:1000
Rabbit monoclonal anti phosphoAkt (Ser 473/Thr 308) antibody	Cell Signaling Technologies	Catt 9271/13038S	polyclonal/Monoclonal antibodies	Rabbit	N/A	N/A	1:1000
Rabbit monoclonal anti-Akt antibody	Cell Signaling Technologies	Catt 9272	Monoclonal primary	Rabbit	N/A	N/A	1:1000
Rabbit monoclonal phospho p70S6Kinase antibody	Cell Signaling Technologies	Catt 9234	Monoclonal primary	Rabbit	N/A	N/A	1:1000
Rabbit monoclonal p70S6 Kinase antibody	Cell Signaling Technologies	Catt 2708	Monoclonal primary	Rabbit	N/A	N/A	1:1000
Rabbit monoclonal anti-Caveolin-1 antibody	Cell Signaling Technologies	Catt 3267	Monoclonal primary	Rabbit	N/A	N/A	1:1000
Digoxin	Sigma-Aldrich	Catt 20830-75-5	N/A	N/A	N/A	N/A	N/A
PP2	Sigma-Aldrich	Catt 172889-27-9	N/A	N/A	N/A	N/A	N/A
Wortmannin	Selleckchem.com	Catt.S2758	N/A	N/A	N/A	N/A	N/A
AG 490	Tocris Bioscience	Catt 0414	N/A	N/A	N/A	N/A	N/A
Goat anti-Mouse IgG1 Cross-Adsorbed Secondary Antibody, Alexa Fluor 488	Thermofischer Scientific	Catt A-21121	Polyclonal Secondary	Goat	N/A	1:100	N/A
FoxO1 (C29H4) Rabbit mAb	Cell Signaling Technologies	Catt2880S	Monoclonal primary	Rabbit	N/A	1:100	1:500
Anti-rabbit IgG (H+L), F(ab')2 Fragment (Alexa Fluor® 488 Conjugate)	Cell Signaling Technologies	Catt 4412	Polyclonal Secondary	Goat	N/A	1:500	N/A
Anti-FOXO3A IgG (phospho S253)	Abcam	Catt ab47285	Polyclonal primary	Rabbit	N/A	1:100	1:500
FoxO3a (75D8) Rabbit mAb	Cell Signaling	Catt 2497S	Monoclonal primary	Rabbit	N/A	1:100	N/A
Anti-Lamin B1 antibody	Abcam	Catt ab16048	Polyclonal primary	Rabbit	N/A	N/A	1:5000
Rabbit IgG HRP-conjugated Antibody	R&D systems	Catt HAF008	Polyclonal Secondary	Goat	N/A	N/A	1:2000
